# Etanercept alleviates psoriasis by reducing the Th17/Treg ratio and promoting M2 polarization of macrophages

**DOI:** 10.1002/iid3.734

**Published:** 2022-11-07

**Authors:** Xiaoqing Li, Ming Jiang, Xia Chen, Weiguo Sun

**Affiliations:** ^1^ Department of Dermatology The Affiliated Huaian No.1 Peoples Hospital of Nanjing Medical University Huai'an Jiangsu P.R. China; ^2^ Department of Pathology The Affiliated Huaian No.1 Peoples Hospital of Nanjing Medical University Huai'an Jiangsu P.R. China

**Keywords:** JAK/STAT3 signaling pathway, macrophage, Netanercept, psoriasis, Th17/Treg

## Abstract

**Introduction:**

This study aimed to investigate the effect of etanercept in psoriasis and its underlying mechanism.

**Methods:**

Female mice were treated with imiquimod (IMQ) to induce psoriasis, and intraperitoneally administered etanercept (0.1–0.4 mg/ml). The RAW264.7 cells were treated with LPS and IFN‐γ to polarize to M1, and were treated with IL‐13 and IL‐4 to polarize to M2.

**Results:**

In our study, Etanercept markedly reduced the psoriasis area and severity index scores, and epidermal thickness of mice induced by IMQ. In addition, etanercept reduced the levels of TNF‐α and IL‐6/12/23, and enhanced the levels of IL‐4/10, reduced Th17/Treg ratio and facilitated the polarization of macrophages to M2 in psoriasis model mice. Furthermore, etanercept inhibited the JAK/STAT3 pathway and increased the protein levels of SOCS1 and SOCS3.

**Conclusions:**

In conclusion, our findings indicated that etanercept could inhibit the JAK/STAT3 pathway to reduce Th17/Treg ratio and promote M2 polarization, thereby alleviating psoriasis of mice.

## Graphical abstract

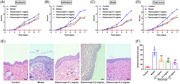

## 1 INTRODUCTION

Psoriasis is a common systemic skin disease caused by the production of inflammatory mediators by macrophages, dendritic cells, and T lymphocytes.^1^ The characteristics of psoriasis include abnormal epidermal differentiation, hyperproliferation, and immune cell infiltration.^2^, ^3^ The typical type of psoriasis is well‐defined, red, scaly plaques of the size as the palm covered by silvery scales, occurring in the scalp, trunk, elbows, and knees.^4^, ^5^ Studies have reported that severe psoriasis is associated with systemic inflammation and comorbidities, including cardiovascular disease, psoriatic arthritis, depression, and diabetes.^6^, ^7^, ^8^, ^9^ Therefore, exploring the mechanism of the occurrence and development of psoriasis is helpful to find effective targets and therapeutic drugs.

Etanercept, an effective inhibitor for TNF‐α, is approved by FDA and EMA for the treatment of arthritis and ankylosing spondylitis.^10^, ^11^, ^12^ Li et al.^13^ have demonstrated that etanercept could reduce the levels of pro‐inflammatory cytokines IL‐1, IL‐6, and LIF to protect rat cardiomyocytes. Study of Tobinick et al. has suggested that perispinal administration of etanercept alleviates cognitive dysfunction, neuropathic pain and microglial activation of patients with stroke and traumatic brain injury (TBI).^14^ Etanercept can promote macrophages towards M2 type polarization in mice with spinal cord injury (SCI) to protect survival motor neurons, reduce injured areas at central lesion sites and significantly improve locomotor recovery.^15^ Recent studies have indicated that etanercept enhances the efficacy of methotrexate (MTX) on patients with psoriatic arthritis (PsA).^16^, ^17^ Etanercept‐mediated modulation of inflammation as a promising approach to reduce white matter injury caused by sepsis or necrotizing enterocolitis in preterm infants.^18^ In addition, Johnston et al. has suggested that etanercept might reduce the tissue responsiveness to IL‐17A by inhibiting the IL17RC expression in keratinocytes.^19^ However, the mechanisms of etanercept on psoriasis have not been fully explored.

In this study, we explored the effect of etanercept on psoriasis progression and its underlining mechanism. Our findings suggested that etanercept alleviated the hyperkeratotic epidermis and abundant inflammatory infiltrates of psoriasis mice. Further studies indicated that etanercept decreased the proportion of Th17 in CD4^+^ T cells and Th17/Treg ratio, and promoted macrophages polarize to M2. We speculated that the effect of etanercept on psoriasis may be achieved through inhibition JAK/STAT3 signaling pathway. Our findings may provide novel insight for the role of etanercept in psoriasis.

## 2 METHODS

### 2.1 Psoriasis mice model induced by imiquimod (IMQ)

The 50 female BALB/c mice (6–8 weeks, 18–20 g, Beijing HFK Bioscience Co., Ltd.) were housed in cages with hardwood chip bedding within pathogen‐free facilities maintained at 20–26°C with a 40%–70% relative humidity and a 12‐hr light:dark cycle. All the mice were fed in standard rodent chow and filtered tap water. Mice were randomly divided into five groups (*n* = 10/per group): Control, Model, Etanercept 0.1 mg/ml, Etanercept 0.2 mg/ml, and Etanercept 0.4 mg/ml (10 mice per group). Vaseline was applied to Control mice. Model mice received 5% Imiquimod (IMQ) cream (Sichuan Med‐Shine Pharmaceutical Co., Ltd.) on the shaved area on the back for 7 days (62.5 mg/d) to induce psoriasis‐like skin inflammation. The Etanercept mice received 5% IMQ cream on the shaved area and 0.1 (0.2 and 0.4) mg/ml etanercept (AbMole Bioscience Inc.) by administrating subcutaneously for 7 days. The mice were randomly allocated into five groups using RANDBETWEEN function in Microsoft Excel.

### 2.2 Psoriasis area and severity index (PASI)

The changes in the skin of mice models were recorded by using the PASI criteria. Erythema, infiltration and scales in each sample of the skin were recorded as 0 (not present), 1 (mild), 2 (moderate), 3 (severe) or 4 (extremely severe) according to the severity, separately. The three index scores were added together to obtain a total score. The trend line for scores of the skin lesion was obtained using the average value of scores in each group.

### 2.3 Collection of samples

After 7 days of modeling, mice were anesthetized to collect peripheral blood samples and skin samples. The peripheral blood samples were centrifugated to obtain serum and then stored at −80°C for the further study. The skin samples were collected for Hematoxylin and eosin (H&E) staining and western blot.

### 2.4 H&E staining

The dorsal skins of mice was collected and fixed in 4% paraformaldehyde in PBS, following by embedding in paraffin. The paraffin‐embedded skins were cut into 5 μm‐thick sections by a slicer and then stained with H&E for histological analysis. The histological changes and the epidermal thickness of skins were observed and measured under a microscope.

### 2.5 Measurement of inflammatory cytokines

The levels of inflammatory cytokines (TNF‐α, IL‐6, IL‐12, IL‐23, IL‐4, and IL‐10) were detected by ELISA and qRT‐PCR. The serum levels of inflammatory cytokines were measured by ELISA kits were performed according to the instructions. The absorbance was read at 450 nm with a microplate spectrophotometer (HACH). The total RNAs of skin samples were isolated using the RNeasy Mini Kit (Qiagen). The mRNA was then transcribed to cDNA. qRT‐PCR was performed on a real‐time PCR system using SYBR Green Premix Ex Taq (TaKaRa). GAPDH was used as the internal control. The relative quantification of genes was calculated by using the $2^{‐∆∆C_{t}}$ method relative to the internal control.

QRT‐PCR was also used to measure the expression levels of M1 phenotype markers (cluster of differentiation [CD] 68 and inductible nitric oxide synthase [iNOS]) and M2 phenotype markers (CD206 and Arginase‐1 [Areg‐1]). The primers were shown in Table 1.

**Table 1:** Primer sequences of genes

Gene	Primer sequence
TNF‐α forward	5′‐TGCTCCТСACCCACACCAT‐3′
TNF‐α reverse	5′‐GCCCAGACTCGGCAAAGTC‐3′
IL‐6 forward	5′‐TGTAGCATGGGCACCTC‐3′
IL‐6 reverse	5′‐CAGTGGACAGGTTTCTGAC‐3′
IL‐12 forward	5′‐CCTCTTCATCCTTACCCC‐3′
IL‐12 reverse	5′‐CACATTGCTCITTCCACCA‐3′
IL‐23 forward	5′‐AAGGGCAAGGACACCATTATTA‐3′
IL‐23 reverse	5′‐CTCCAGGCTTCTCACAGTTTCT‐3′
IL‐4 forward	5′‐ТСТССТСССССАСТГСТАСА‐3′
IL‐4 reverse	5′‐GTCGAGCCGTTTCAGGAATC‐3′
IL‐10 forward	5′‐TACAGCCGGGAAGACAATAA‐3′
IL‐10 reverse	5′‐AGGAGTCCGTTAGCAGTATG‐3′
CD68 forward	5′‐GGTTGAGGAAGGAGCTGTTACAGCC‐3′
CD68 reverse	5′‐GGCTGTAACAGCTCCTTCСTСAACC‐3′
iNOS forward	СТССАССАСТТСGАТСAСGAAСCТG‐3′
iNOS reverse	5′‐CGAGTACCCTGTCTGCACCTGGAA‐3′
CD206 forward	5′‐TTCGGACACCCATCGGAATTT‐3′
CD206 reverse	5′‐CACAAGCGCTGCGTGGAT‐3′
Arg‐1 forward	5′‐GGAAATCGTGGAAATGAG‐3′
Arg‐1 reverse	5′‐CAGATATGCAGGGAGTCACC‐3′
GAPDH forward	5′‐CCCACTCCTCCACCTTTGAC‐3′
GAPDH reverse	5′‐ATGAGGTCCACCACCCTGTT‐3′

### 2.6 Analysis of Treg and Th17 cell proportion

The cell proportion of Treg and Th17 in T cells was analyzed by flow cytometry.^20^, ^21^ To analyze the Treg and Th17 cells, blood samples (3 ml) were added to RPMI 1640 medium and activated by leukocytes. The mixture was incubated 4 h at 37°C with 5% CO_2_, and then was placed in centrifuge tube and mixed with CD4‐FITC at 4°C for 15 min in the dark condition. Next, centrifugated the cell suspension and discarded the supernatant, following by washing in staining buffer. The Perm/Fix solution was used to fix and permeabilize cells. Subsequently, the cells were centrifuged and re‐suspended in PBS containing both allophycocyanin (APC)‐conjugated anti‐mouse Foxp3‐APC and phycoerythrin (PE)‐cy7‐conjugated anti‐mouse IL‐17 antibody (eBioscience) using manufacturer recommended dilutions. After incubation at 4°C for 30 min in the dark, the cells were then gently washed with PBS and analyzed in a BD Accuri™ C6 Plus flow cytometer (BD) using FlowJo software (Treestar). A minimum of 30,000 events (CD4 + lymphocytes) per sample was acquired (by CD4 + gating); each sample was analyzed in triplicate. From the total numbers of both TH17 and Treg cells in each sample, TH17/Treg ratios were calculated.

### 2.7 Macrophage polarization

RAW264.7 cells (immortalized murine macrophage cell line) were pretreated with LPS (100 ng/ml) and IFN‐γ (10 ng/ml) for 6 h to polarize to M1 macrophages, and pretreated with IL‐4 (20 ng/ml) and IL‐13 (20 ng/ml) to polarize to M2 macrophages.

### 2.8 Western blot assay

The total protein of skin tissues was extracted by using radioimmunoprecipitation assay (RIPA) lysis buffer containing phosphatase inhibitors and a protease inhibitor mixture at 4°C for 1 h, following by centrifuging for 10 min at 4°C. The supernatant was collected and the protein concentration was determined by the BCA Kit. Then, the samples were separated by SDS‐PAGE gels, and were transferred onto PVDF membrane. The membranes were incubated with 5% nonfat dry milk for 1 h at 37°C, and then incubated with primary antibodies (p‐JAK, JAK, p‐STAT3 and STAT3 were purchased from Abcam) overnight at 4°C. Then the membranes were washed three times using TBST and incubated with secondary antibody for 1 h. The intensity of protein band was quantified using Image J.

### 2.9 Statistical analysis

Data in this study were analyzed by using GraphPad Prism 7.0 (GraphPad) and presented as mean ± SD. The comparisons among different groups were performed using ANOVA, followed by Tukey's post hoc test to compare each pair of columns. A *p* < .05 was accepted as significant.

## 3 RESULTS

### 3.1 Etanercept improves psoriasis‐like lesions of psoriasis model mice

**Figure 1:**
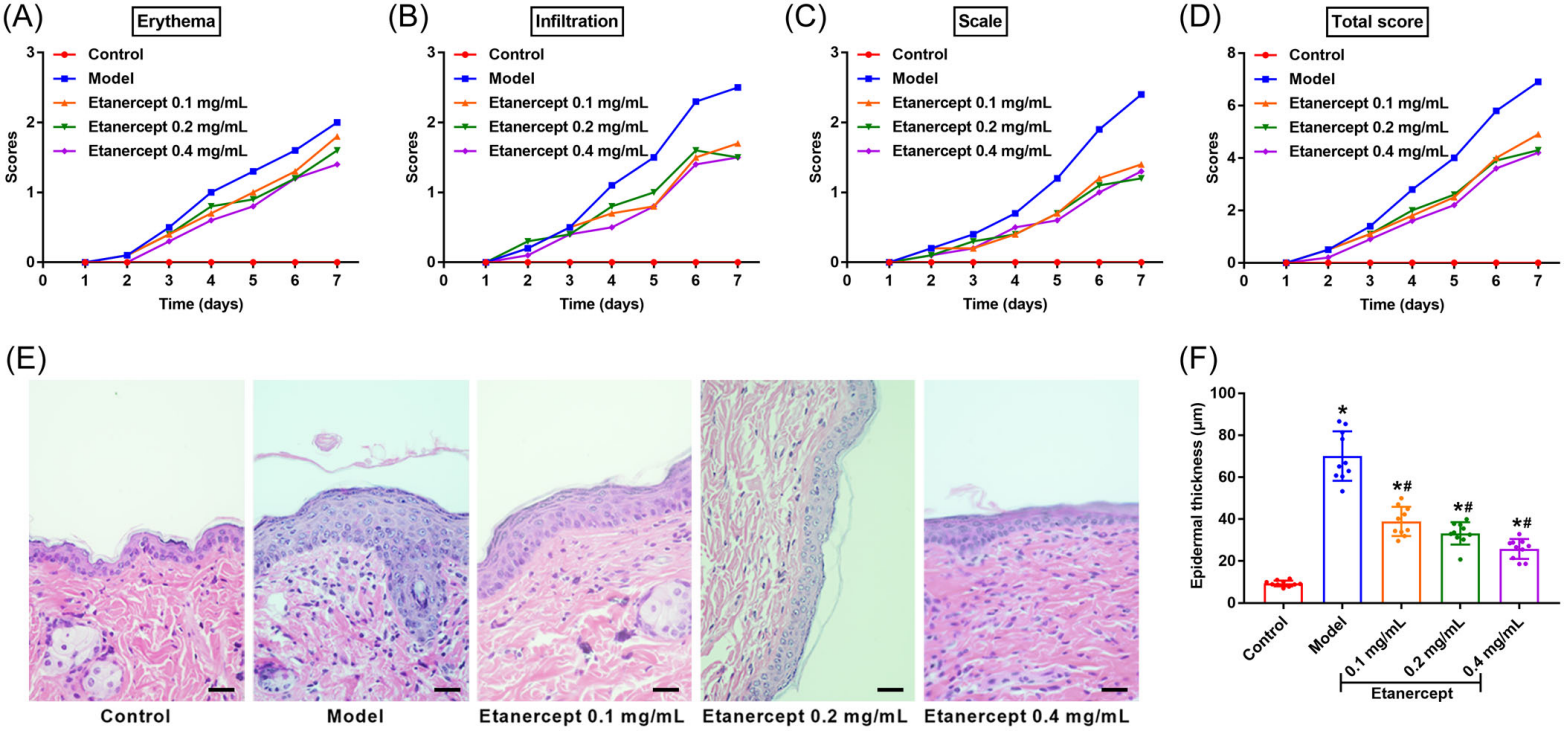
The pathological changes of psoriasis induced by IMQ in mice. (A/B/C/D) The skin changes of mice (Control, Model, Etanercept (0.1 mg/ml), Etanercept (0.2 mg/ml) and Etanercept (0.4 mg/ml) groups) recorded by PASI scores. (E) The skin changes of mice detected by H&E staining (×400). Scale bar = 50 μm. (F) The changes of epidermal thickness were measured by the image analysis system. *p < .05 compared with Control group; #*p* < .05 compared with Model group. IMQ, imiquimod; PASI, Psoriasis area and severity index.

We established mice model of psoriasis by applying IMQ on the shaved area on the back of mice and recorded the changes in the skin of mice models were recorded by using the PASI criteria. The PASI results indicated that The scores of erythema, infiltration, scale, and total score in Etanercept groups were dramatically decreased in comparison with the Model group (Figure 1A–D, *p* < .05). Besides, we observed the histopathological changes and epidermal thickness by the H&E staining. The results suggested that the mice of Model group showed hyperkeratotic epidermis, while skins of etanercept mice showed less hyperkeratosis and epidermal thickening (Figure 1E). The data in Figure 1F also indicated that the etanercept treatment significantly reduced the epidermal thickness of Model mice skin (*p* < .05).

### 3.2 Etanercept decreases inflammatory levels of psoriasis model mice

**Figure 2:**
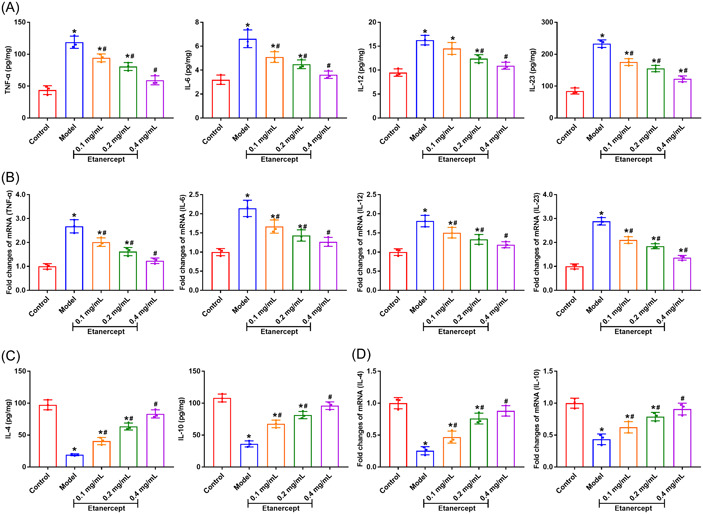
Etanercept decreases inflammatory levels of psoriasis mice. (A/B) The levels of pro‐inflammatory cytokines TNF‐α, IL‐6, IL‐12, and IL‐23 in the skin of mice were measured by ELISA and qRT‐PCR, respectively. (C/D) The levels of anti‐inflammatory cytokines IL‐4 and IL‐10 in the skin of mice were measured by ELISA and qRT‐PCR, respectively. *p < .05 compared with Control group; #*p* < .05 compared with Model group.

We detected the inflammatory levels in IMQ induced mice by ELISA and qRT‐PCR. The levels of pro‐inflammatory cytokines (TNF‐α, IL‐6, IL‐12, and IL‐23) were dramatically increased in mice of Model group in comparison with Control group (Figure 2A,B, *p* < .05), while the levels of anti‐inflammatory cytokines (IL‐4 and IL‐10) in Model group were markedly decreased (Figure 2C,D, *p* < .05). In comparison with the Model group, the levels of TNF‐α, IL‐6, IL‐12, and IL‐23 were dramatically reduced after the etanercept treatment (Figure 2A,B *p* < .05). The levels of IL‐4 and IL‐10 in Etanercept groups was markedly increased compared to the Model group (Figure 2C,D, *p* < .05).

### 3.3 Etanercept reduces the ratio of Th17/Treg in peripheral blood of psoriasis model mice

**Figure 3:**
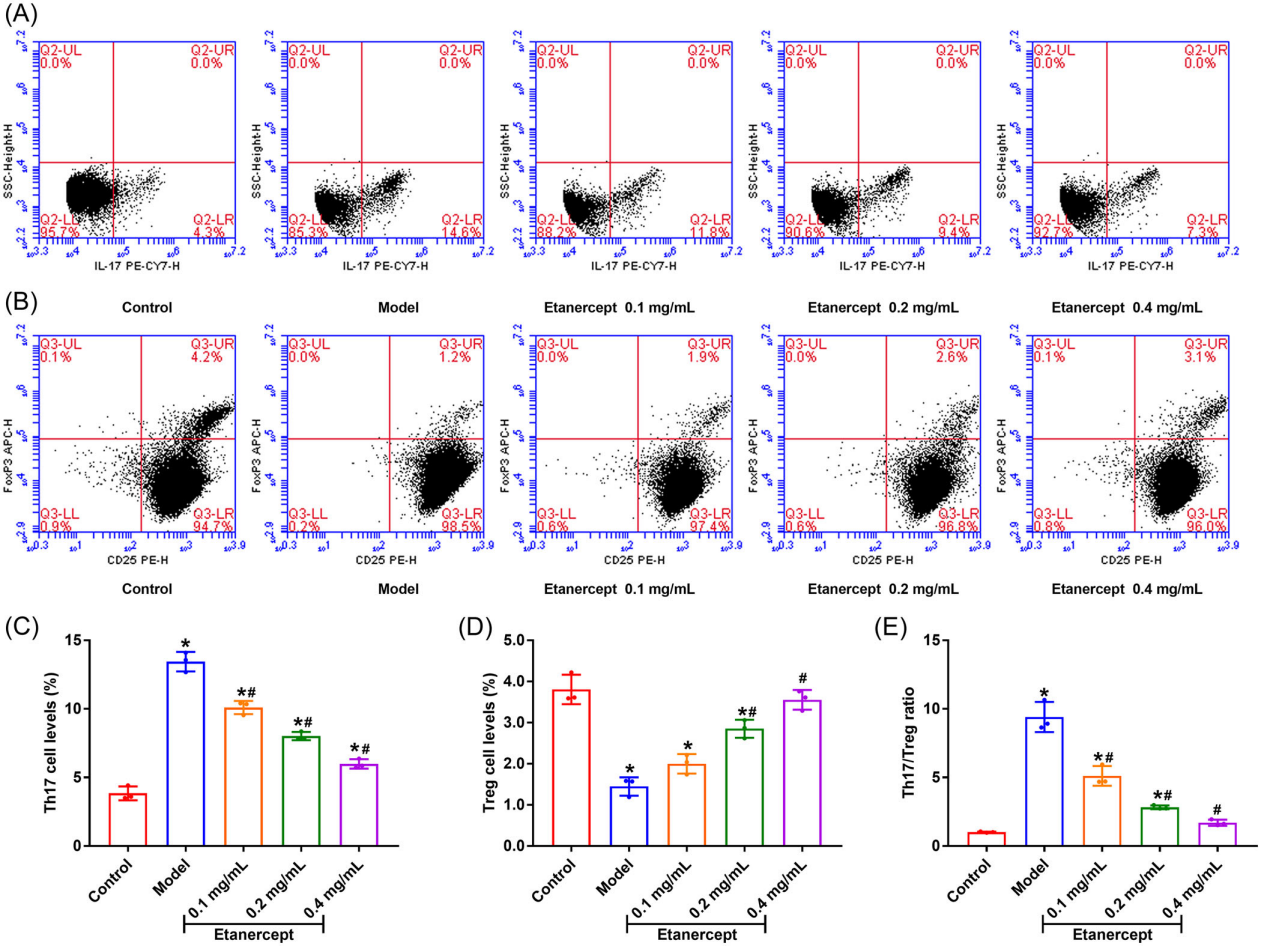
Etanercept reduces the ratio of Th17/Treg in psoriasis mice. (A/C) The Th17 levels of mice in peripheral blood were detected by flow cytometry. (B/D) The Treg levels of mice in peripheral blood were detected by flow cytometry. (E) The changes of Th17/Treg ratio. *p < .05 compared with Control group; #*p* < .05 compared with Model group.

We analyzed the proportions of Th17/Treg proportion in CD4^+^ T cells to investigate the influence of etanercept on T cells. As shown in Figure 3A,B, THE Th17 cell proportion in peripheral blood was dramatically increased in the Model group in comparison with Control group (*p* < .05). The Treg cell proportion in peripheral blood was remarkably lower in Model group than that in Control group (Figure 3B,D, *p* < .05). Th17/Treg ratio was remarkably raised in the Model group in comparison with the Control group (Figure 3E, *p* < .05). Etanercept treatment reduced Th17 cell proportion and increased Treg proportion of psoriasis mice with dose dependent effect (Figure 3A–D). The ratios of Th17/Treg in mice with etanercept treatment were showed dose‐depended decrease compared with Model group (Figure 3E). The above results indicated that etanercept reduced Th17 proportion, increased Treg proportion and reduced the Th17/Treg ratio.

### 3.4 Etanercept promotes M2 polarization of macrophages in psoriasis model mice

**Figure 4:**
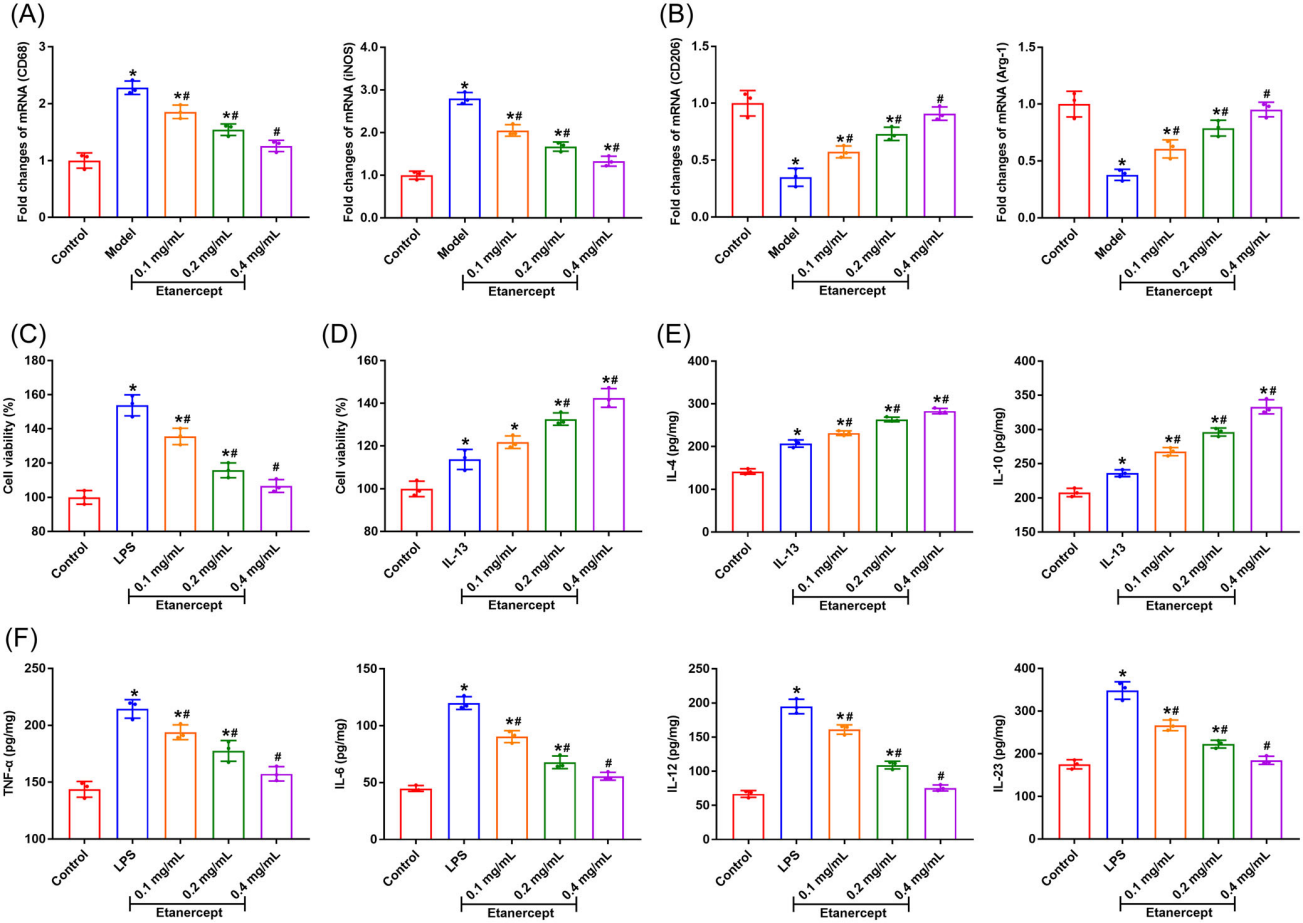
Etanercept promotes M2 polarization of macrophages in psoriasis mice. (A) The expression levels of M1 phenotype markers CD 68 and iNOS were detected by qRT‐PCR. (B) The expression levels of M2 phenotype markers CD206 and Areg‐1 were detected by qRT‐PCR. (C/D) The cell viability of M1/M2 macrophages was detected by CCK‐8 assay. (E) The levels of anti‐inflammatory cytokines IL‐4 and IL‐10 in M2 macrophages were detected by ELISA. (F) The levels of pro‐inflammatory cytokines TNF‐α, IL‐6, IL‐12, and IL‐23 in M1 macrophages were detected by ELISA. *p < .05 compared with Control group; #*p* < .05 compares with Model group.

We measured the expression of M1/M2 phenotype markers in peripheral blood samples to detect the effect of etanercept on macrophages in psoriasis model mice. The data in Figure 4A,B suggested that the expression of M1 phenotype markers CD68 and iNOS were significantly enhanced in psoriasis mice, while the expression of M2 phenotype markers CD206 and Arg‐1 were dramatically decreased (*p* < .05). We stimulated RAW264.7 macrophage with LPS and IL‐13 to polarize to M1 and M2, respectively. Then, we detected the effect of etanercept on cell viability of M1/M2 macrophages by CCK‐8 assay. The data showed that etanercept treatment decreased the cell viability of M1 macrophages and increased the cell viability of M2 macrophages dose‐dependently (Figure 4C,D, *p* < .05). Besides, we detected the expression levels of inflammatory cytokines in M1/M2 macrophages. The data showed that etanercept treatment increased the levels of IL‐4 and IL‐10 in M2 macrophages, and reduced levels of TNF‐α, IL‐6, IL‐12, and IL‐23 in M1 macrophages (Figure 4E,F, *p* < .05). These findings showed that etanercept promotes macrophages polarize to M2 in psoriasis model mice.

### 3.5 Etanercept inhibits the JAK/STAT3 signaling pathway of psoriasis model mice

**Figure 5:**
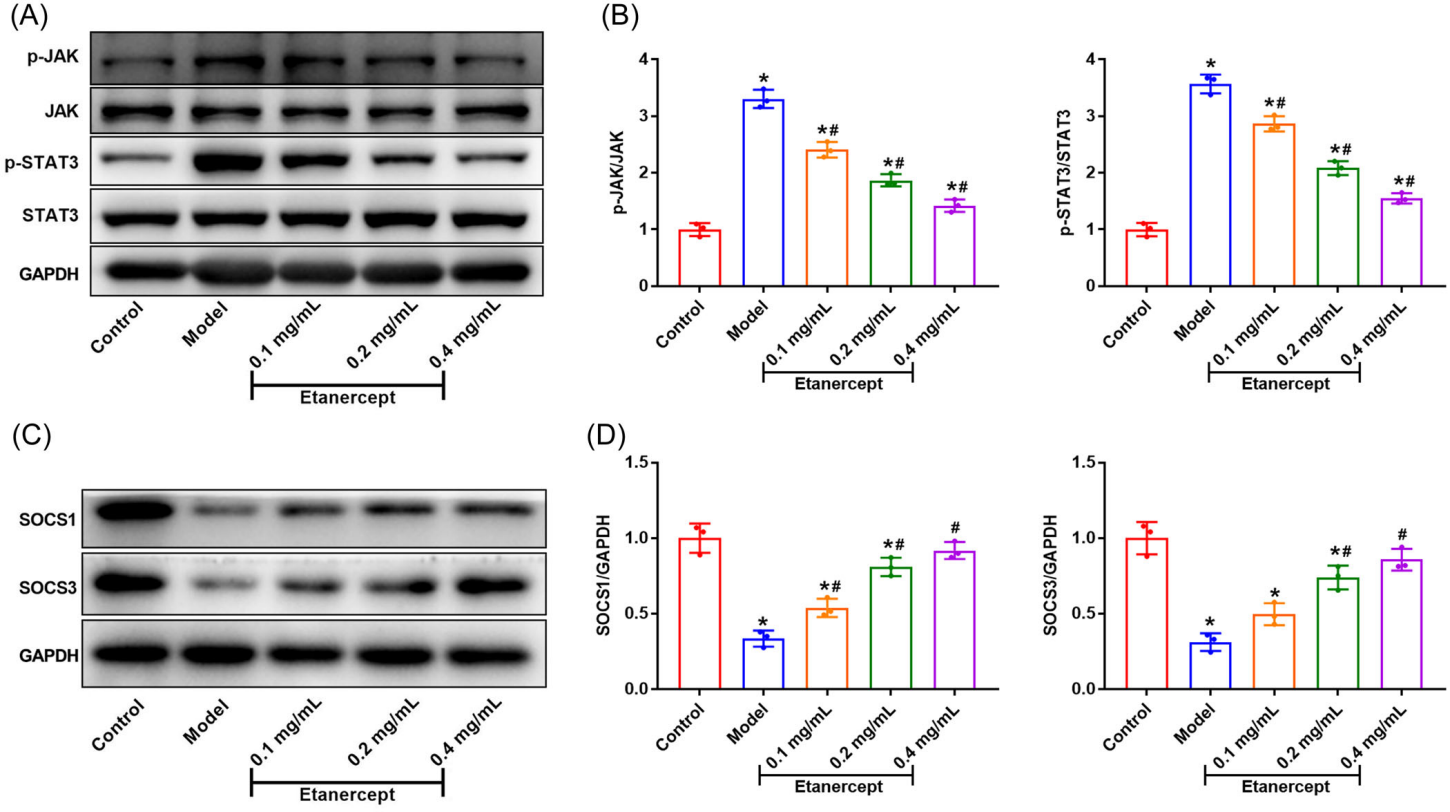
Etanercept inhibits the JAK/STAT3 signaling pathway of psoriasis model mice. (A/B) The protein expression of p‐JAK, JAK, p‐STAT3, and STAT3 was detected by western blot. (C/D) The protein expression of SOCS1 and SOCS3 was detected by western blot. *p < .05 compares with Control group; #*p* < .05 compared with Model group.

To investigate the mechanisms of etanercept on IMQ–induced psoriasis mice, we detected the expression of JAK/STAT3 pathway. The results showed that the protein expression of p‐JAK/JAK and p‐STAT3/STAT3 in Model group was significantly enhanced compared with Control group (Figure 5A,B, *p* < .05), indicating that psoriasis modeling by IMQ activated JAK/STAT3 signaling pathway. Compared with Model group, etanercept treatment significantly decreased the protein expression of p‐JAK/JAK and p‐STAT3/STAT3 (*p* < .05), and the inhibitory effect of etanercept on JAK/STAT3 signaling pathway was dose dependent.

Additionally, we carried out western blot to detect the expression of SOCS1 and SOCS3. Compared to the control group, the expression of SOCS1 and SOCS3 in psoriasis mice was significantly decreased (Figure 5C,D, *p* < .05). Etanercept treatment enhanced the protein expression of SOCS1, and the expression of SOCS1 and SOCS3 increased with the increase of etanercept dose. These above results suggested that etanercept inhibited JAK/STAT3 signaling pathway and protein expression of SOCS1 and SOCS3.

## 4 DISCUSSION

Psoriasis is a noncommunicable skin disease that can occur in people of all ages.^22^ The worldwide prevalence of psoriasis is about 2% and the prevalence is up to 11% in some areas, making psoriasis a serious global problem.^23^ Currently, the main drugs for the therapy of psoriasis are small‐molecule therapies (MTX, cyclosporin A and retinoids) and biological agents (infliximab, adalimumab and etanercept).^24^, ^25^, ^26^ In this study, we studied the effect of etanercept in psoriasis and its potential mechanism. The results showed that etanercept reduced the PASI score, reduced epidermal thickening, and decreased inflammatory levels of psoriasis mice. We found etanercept could decrease the Th17/Treg ratio in CD4^+^ T cells and induce macrophages polarize to M2 in psoriasis mice. Additionally, etanercept inhibited the activation of JAK/STAT3 signaling pathway and promoted the protein expression of SOCS1 and SOCS3 in psoriasis mice.

Th17 cells are characterized by IL‐17 production and play a critical role in the induction of autoimmune tissue injuries and inflammation.^27^ The number of Th17 cells is elevated in active uveitis and scleritis and decreased after treatment.^28^ Regulatory T cells could suppress or release cytokines to play an anti‐inflammatory role, which is essential for maintaining immunological self‐tolerance and immune homeostasis.^29^, ^30^ Studies have indicated that the imbalance of Th17/Treg is associated with different inflammation and autoimmune diseases.^31^, ^32^, ^33^ Predecessors' research has demonstrated that the imbalance between Th17/Treg is an essential reason for rheumatoid arthritis (RA).^34^ In this study, psoriasis modeling significantly increased the ratio of Th17/Treg in CD^+^ T cells of mice. Etanercept treatment reduced Th17 proportion and increased Treg proportion in CD4^+^ T cells, thereby decreased Th17/Treg ratio in in CD4^+^ T cells dose‐dependently. Besides, etanercept reduced pro‐inflammatory cytokines levels and increased anti‐inflammatory cytokines levels of psoriasis mice. These results indicated that etanercept might alleviate psoriasis by regulating Th17/Treg ratio and expression of inflammatory cytokines.

Macrophages play an important role in many inflammatory diseases, including diabetes, periodontitis and other inflammatory diseases.^35^, ^36^, ^37^ It is accepted that macrophage activation leading to two polarized states: the M1 and M2 phenotypes.^38^ Gwak et al. found that heme oxygenase‐1 played a key role in the resolution of experimentally induced colitis by modulating the polarization of macrophages.^39^ Suppressing M1 markers and augmenting M2 polarization of microglia exhibits neuroprotective effects in the postischemic brain.^40^ In this study, we found that the expression of M1 markers (CD68 and iNOS) was enhanced in psoriasis mice and the pro‐inflammatory cytokines levels (TNF‐α, IL‐6, IL‐12, and IL‐23) were increased. The expression of M2 markers (CD206 and Arg‐1) was reduced and the levels of anti‐inflammatory cytokines (IL‐4 and IL‐10) were decreased. Etanercept suppressed the expression of M1 markers and the levels of pro‐inflammatory cytokines, as well as increased the expression of M2 markers and levels of anti‐inflammatory cytokines. The above data suggested that etanercept might remit psoriasis by promoting the polarization of macrophages to M2 in psoriasis model mice.

The JAK/STAT pathway is a simple pathway that could be activated by inflammatory cytokines.^41^ Ni et al.^42^ have indicated that chemical inhibitor targeted to JAK/STAT3 pathway retarded the RA progression and ameliorate RA symptoms. Inhibition of the JAK/STAT3 pathway promoted the activation of microglia to the M2 phenotype and enhanced the phagocytic function of microglial BV‐2 cells.^43^ Moreover, Suppression of STAT3 phosphorylation inhibited pro‐inflammatory cytokine/chemokine production in mice of IMQ‐induced psoriatic dermatitis.^44^ Additionally, incubation of glia with TNF‐α induced the phosphorylation of JAK2 and STAT1 and the interaction of JAK2 with the TNF‐α receptor (TNFR1).^45^ Following the activation of JAK2 kinases, STAT3 can act as a transcription factor to trigger the expression of various pro‐inflammatory cytokines, including TNF‐α.^46^ Presently, the results in our study showed that the protein expression of p‐JAK/JAK and p‐STAT3/STAT3 was sharply increased in psoriasis mice, and etanercept treatment blocked the expression of p‐JAK/JAK and p‐STAT3/STAT3. Suppressor of cytokine signaling (SOCS) proteins are key anti‐inflammatory regulators of JAK‐STAT pathway.^47^ As important members of the SOCS family, SOCS1 and SOCS3 play a key role in regulating inflammatory expression.^48^ We found that the protein expression of SOCS1 and SOCS3 was significantly decreased in model mice, and etanercept enhanced the protein expression of SOCS1 and SOCS3 in a dose‐depended manner. These results suggested that etanercept might remit psoriasis by inhibiting the JAK/STAT3 signaling pathway and promoting the protein expression of SOCS1 and SOCS3.

In summary, we found that etanercept could alleviate the symptoms of psoriasis of IMQ‐induced mice, and the underlying mechanism was to inhibit the JAK/STAT3 signaling pathway and enhance the expression of SOCS1 and SOCS3, thereby decreasing the expression of inflammatory cytokines, reducing Th17/Treg ratio and promoting the polarization of macrophages to M2. However, this study is associated with certain limitations. This study lacks clinical evidence that etanercept can play a specific role in regulating inflammation, macrophages polarization and Th17/Treg ratio in psoriasis, which is also the focus of our future research. Greater sample size and involvement of more study centers are required to verify this result. Additionally, etanercept is an antibody against tumor necrosis factor, so its mechanism of action may be related to affecting Th1 cell function. However, this study lacks the detection of Th1 cell function. This part should also be included in future study.

## AUTHOR CONTRIBUTIONS

Xiaoqing Li designed the study; Ming Jiang and Xia Chen obstained data, Xiaoqing Li and Weiguo Sun analyzed data; Xiaoqing Li wrote the paper.

## CONFLICT OF INTEREST

The authors declare no conflict of interest.

## ETHICS STATEMENT

The experimental protocol of our study was performed in accordance with the Guide for the Care and Use of Laboratory Animals and approved by The Affiliated Huaian No.1 Peoples Hospital of Nanjing Medical University.

## Data Availability

The data sets used and analyzed during the current study are available from the corresponding author on reasonable request.
